# Small Molecule Tyrosine Kinase Inhibitors (TKIs) for Glioblastoma Treatment

**DOI:** 10.3390/ijms25031398

**Published:** 2024-01-23

**Authors:** Davide Frumento, Giancarlo Grossi, Marta Falesiedi, Francesca Musumeci, Anna Carbone, Silvia Schenone

**Affiliations:** Department of Pharmacy, University of Genoa, Viale Benedetto XV 3, 16132 Genoa, Italy; davide.frumento@edu.unige.it (D.F.); giancarlo.grossi@unige.it (G.G.); marta.falesiedi@edu.unige.it (M.F.); silvia.schenone@unige.it (S.S.)

**Keywords:** glioblastoma multiforme, tyrosine kinase inhibitors, brain cancers, small molecules, clinical trials

## Abstract

In the last decade, many small molecules, usually characterized by heterocyclic scaffolds, have been designed and synthesized as tyrosine kinase inhibitors (TKIs). Among them, several compounds have been tested at preclinical and clinical levels to treat glioblastoma multiforme (GBM). GBM is the most common and aggressive type of cancer originating in the brain and has an unfavorable prognosis, with a median survival of 15–16 months and a 5-year survival rate of 5%. Despite recent advances in treating GBM, it represents an incurable disease associated with treatment resistance and high recurrence rates. For these reasons, there is an urgent need for the development of new pharmacological agents to fight this malignancy. In this review, we reported the compounds published in the last five years, which showed promising activity in GBM preclinical models acting as TKIs. We grouped the compounds based on the targeted kinase: first, we reported receptor TKIs and then, cytoplasmic and peculiar kinase inhibitors. For each small molecule, we included the chemical structure, and we schematized the interaction with the target for some representative compounds with the aim of elucidating the mechanism of action. Finally, we cited the most relevant clinical trials.

## 1. Introduction

Glioblastoma (GBM) is an intrinsic brain cancer originating from neuroglial stem or progenitor cell anomalies [1]. Under physiological conditions, neural progenitor cells (NPCs) give origin to central nervous system (CNS) glial and neuronal cell populations [2], while metabolic enzymes-related gene mutations afford pathological transformation in GBM cells [3]. GBM is characterized by a high degree of heterogeneity at the genetic and cellular level, thus it is difficult to treat. For this reason, the identification of new biomarkers constitutes a promising approach to improve diagnosis and to find cellular pathways to target [4]. According to the gliomas classification system, GBM is identified as grade IV lesion found in astrocytic cancer forms [5]. GBM development involves a total of 92 immune-related genes. Interestingly, the 14 most representative genes show an evident correlation with prognosis [6]. GBM has an overall incidence of approximately 240,000 new cases each year and its occurrence is closely tied to age, with the median age at diagnosis falling within the 65-year range, and affecting males approximately 1.7 times more frequently than females [7]. The disease is generally characterized by a poor prognosis with a 10-years survival rate of 0.71% and a median survival of 15 months [8,9]. The current standard of care includes maximal surgical resection, radiotherapy and temozolomide (TMZ) chemotherapy [10].

In addition to TMZ, there are currently three FDA-approved drugs for high-grade glioma (HGGs), i.e., bevacizumab (BVZ), lomustine and intravenous carmustine [11]. BVZ, approved in 2009 to treat recurrent GBM, is a therapeutic antibody binding and inhibiting vascular endothelial growth factor (VEGF). Since malignant gliomas are characterized by a strong neovascularization, BVZ is administered to prevent angiogenesis by VEGF inhibition. Lomustine, also named CCNU (from its chemical name chloroethyl-cyclohexyl-nitrosourea) is a non-specific alkylating agent approved in 1976 to treat HGGs. It is responsible to crosslink DNA and RNA in proliferating cells, thus triggering cell death in cancer cells. Similarly, carmustine, also known as BCNU (bis-chloroethyl-nitrosourea), is a non-specific alkylating agent approved by in 1977 to treat HGGs [11].

Among FDA-approved devices, only carmustine wafer implants and tumor treatment fields (TTFields) were approved for new diagnoses [11]. Carmustine wafer implants were approved for HGGs treatment in 1996 and consist of biodegradable polymer wafers, each containing 7.7 mg of carmustine. TTFields are portable devices applied to the shaved scalp, delivering low-intensity (1–3 V/cm) and intermediate-frequency (200 kHz) alternating electric fields, disrupting mitosis in cancer cells. When compared with the current standard of care, only TTFields improve overall survival (20.5 vs. 15.6 months) and progression-free survival at six months (56% vs. 37%) [11]. Moving towards strategies to sustain a longer response and increase the long-term survival rates, isotretinoin (13-cis retinoic acid) was studied. This compound is a synthetic retinoid inhibiting cell proliferation and migration. It was already evaluated for both sustainment therapy and relapsed disease in HGGs [12,13].

In the immunotherapy field, the use of chimeric antigen receptor (CAR) T cells and immune checkpoint inhibitors are emerging as valuable strategies to tackle GBM. CAR T cell therapy is a relatively recent approach in which T lymphocytes from a patient are genetically modified to target the tumor once reinfused into the patient’s body. For GBM, the antigen-recognition molecules that confer antigen specificity are, for example, epidermal growth factor receptor variant III (EGFRvIII), interleukin (IL)-13R2 and human epidermal growth factor receptor 2 (HER2) [14].

Immune checkpoint inhibitors enhance the body’s natural immune response against cancer cells. Some commonly targeted checkpoint proteins include programmed cell death protein 1 (PD-1), programmed cell death ligand 1 (PD-L1), and cytotoxic T-lymphocyte-associated protein 4 (CTLA-4). Several phase I trials are evaluating these approaches for GBM patients. For instance, phase I trial NCT02794883 investigated the safety of CTLA-4 and PD-L1 antibodies in recurrent GBM patients [15]. Also, Phase I trial NCT02311920 evaluated anti-CTLA-4 and/or anti- PD-1 [16] combined with TMZ for newly diagnosed GBM/gliosarcoma patients.

Protein kinases, one of the largest enzyme families in mammalians [17], exert their catalytic action by transferring the terminal phosphate group from nucleotide triphosphates to serine, tyrosine or threonine OH-groups. Indeed, protein kinases are divided in serine-threonine kinases (STK), tyrosine kinases (TKs) and non-specific TK. Kinases bind ATP in a pocket between the catalytic domain’s lobes, and the interaction is stabilized by multiple hydrogen bonds [18]. Kinases can be classified into transmembrane receptors and, intracellular kinases. In physiological conditions, these enzymes play a pivotal role in cell growth, proliferation, differentiation and apoptosis. These enzymes are overexpressed, hyperactivated or mutated in different types of cancers, included GBM [19]. Kinases represent pharmacologically attractive targets for anticancer treatment due to a high degree of conservation of their catalytic domain, low toxicity and oral availability [20]. Many kinase inhibitors, such as ATP-competitive types I and II, as well as allosteric types III and IV, have been developed in last decades [21].

GBM is characterized by several signaling pathways alterations. Among them, phosphoinositide 3-kinase/protein kinase B (PI3K/AKT) regulatory pathway is one of the most important. In fact, aberrant AKT activation contributes to resistance towards first generation TKIs, such as the EGFR inhibitor gefitinib [22]. Interestingly, 67.3% of GBMs exhibits some kinases mutation or amplification, specifically for EGFR, PDGFR, FGFR2/3 and MET [23].

**Figure 1:**
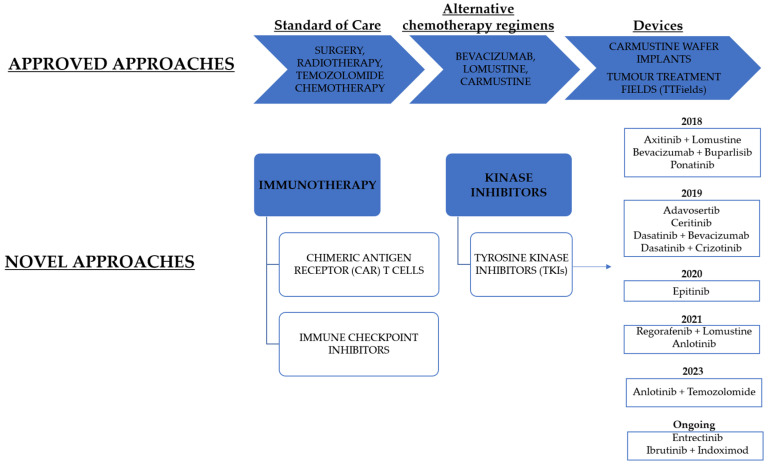
A schematic representation of current and ongoing therapeutic approaches for GBM treatment. TKIs are classified based on the year of the clinical trial completion.

Many kinase inhibitors have been approved to treat several types of cancers but none of them still reached approval, alone or in combination, for GBM therapy. A schematic summary of the current and ongoing therapeutic approaches are reported in the Figure 1. In this review, we report the state-of-the-art of small molecule tyrosine kinase inhibitors (TKIs) tested in different GBM models, focusing on the last five years. The inhibitors are described based on the targeted kinases. Furthermore, the current clinical trials involving TKIs alone or in combination with other agents, are reported.

## 2. TK Inhibitors

### 2.1. ALK Inhibitors

Anaplastic lymphoma kinase (ALK) is a transmembrane receptor tyrosine kinase that plays a central role in neurogenesis [24,25]. Several cancer forms express aberrant ALK variants due to amplifications, point mutations, and chromosomal rearrangements [26,27,28]. Interestingly, ALK is overexpressed in glioblastoma (GBM) [29,30]. The analysis conducted by Karagkounis et al. on ALK expression in 51 GBM patients revealed its presence in most samples, although rearrangements appear to be relatively uncommon [29,31]. ALK receptor represents a starting signal able to stimulate various pathways (e.g., Ras/MAPK, mTOR/PI3K/Akt, phospholipase C-g) [31]. Since abnormal regulation of PI3K/Akt signaling has been found in U87 GBM cells [32], ALK inhibition represents a promising therapeutic option in GBM treatment. ALK inhibitors have been tested alone or in combination with other anticancer agents to treat GBM. Unfortunately, mutated ALK lacks sensitivity to monotherapies with ALK inhibitors and prevents their use as a first-line therapy [33].

#### Crizotinib, Alectinib and Ceritinib

**Figure 2:**
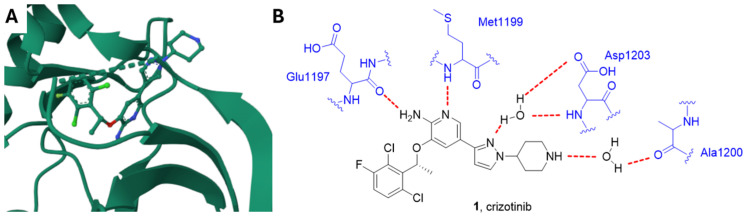
(**A**) Crystal structure of ALK in complex with crizotinib (PDB: 2XP2) [36]. (**B**) Schematic representation of the main interactions between crizotinib and ALK with amino acid residues crucial to the interaction highlighted in blue. Hydrogen bonds are represented as red dashed lines.

**Figure 3:**
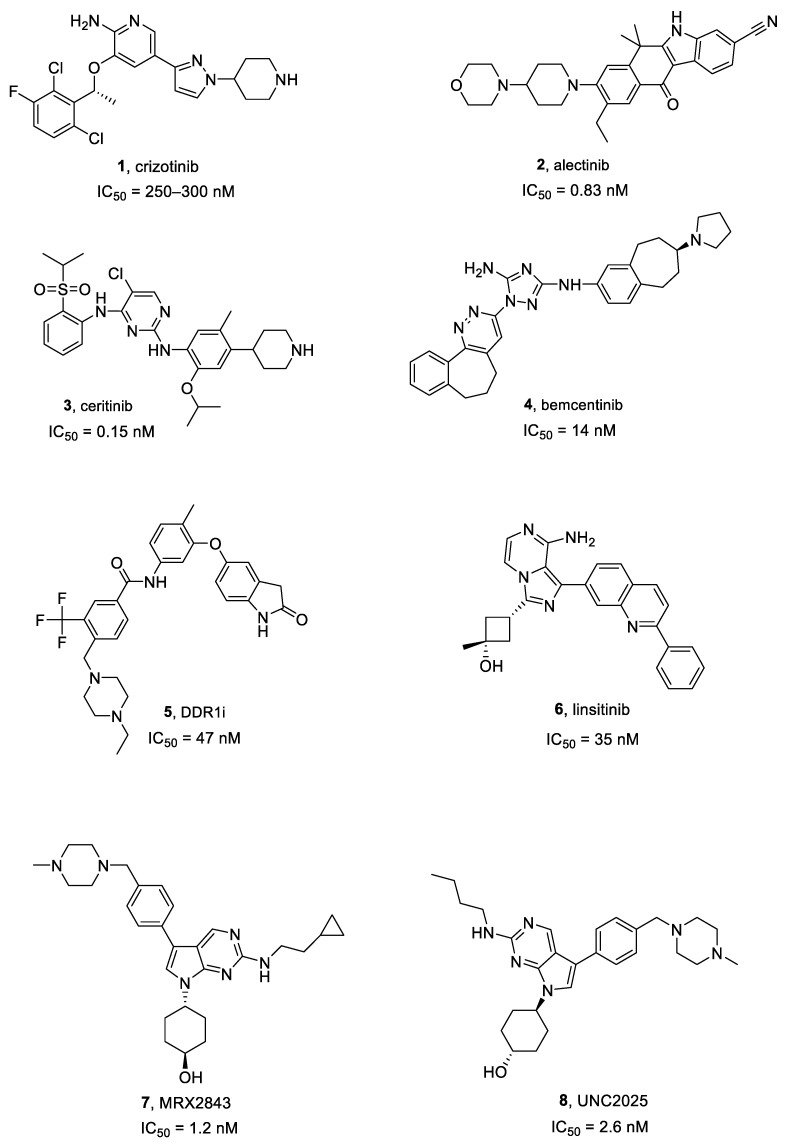
Structures of ALK, AXL, DDR1, IGF1R, and MER inhibitors **1**–**8**.

Crizotinib **1** (Figure 2 and Figure 3) is an approved first generation ALK inhibitor (IC_50_ vs. EML4-ALK = 250–300 nM) [34] used to treat ALK-positive non-small cell lung cancer (NSCLC).

Crizotinib is a type I kinase inhibitor and binds ALK in its active conformation through the formation of different hydrogen bonds. In detail, the NH_2_ group and N1 of the pyridine ring form two hydrogen bonds with the backbone oxygen atom of Glu1197 and the backbone NH hydrogen atom of Met1199, respectively. In addition, two hydrogen bonds between the inhibitor and the catalytic pocket are mediated by two molecules of water; specifically, the pyrazole N2 nitrogen atom forms a hydrogen bond with a water molecule bound to Asp1203, and the NH group of the piperidine interacts with Ala1200 through another water molecule (Figure 2) [35].

During the first two years of administration, resistance phenomena were commonly observed, due to both ALK TK domain mutations and alternative signaling pathway activation. Thus, the development of more potent and structurally different inhibitors emerged as a primary need [34].

Recently, crizotinib in combination with TMZ and standard radiotherapy showed a tolerable safety profile and promising efficacy as a first-line therapy for GBM patients [37].

Alectinib **2** (Figure 3) is a second-generation ALK inhibitor, approved in 2015 for the advanced ALK-positive NSCLC patients who showed disease progression or were intolerant to crizotinib. It is a highly specific ALK blocker (IC_50_ = 0.83 nM) [38,39]. According to Sakamoto et al. [39], alectinib inhibited ALK L1196M, the pivotal mutation commonly conferring resistance to TKIs, and blocked EML4-ALK L1196M-driven cell growth. Clinically, it was also partially effective on NSCLC brain metastasis for 52% of examined patients in a phase I/II study [40]. Alectinib reduced proliferation and induced apoptosis in ALK expressing GMB cells, while cells depleted for ALK or showing a high expression of cMyc and activation of the ERK1/2 pathway led to a primary resistance against alectinib. The inhibition of these pathways using a cMyc inhibitor or a MEK inhibitor, overcame alectinib resistance. The combination of alectinib with radiotherapy induced a synergistic effect in inhibition of proliferation in non-resistant and alectinib resistant GBM cells [41].

Ceritinib **3** (Figure 3), another second-generation ALK inhibitor (IC_50_ = 0.15 nM versus ALK) was approved in 2014 for ALK-positive NSCLC treatment [42]. Interestingly, it is characterized by a high-ALK selectivity, about 20-fold more than crizotinib [42]. Moreover, a multicenter phase II trial showed a 45% intracranial response in NSCLC brain metastasis, based on response evaluation criteria in solid tumors [43].

Kawauchi et al. [44] demonstrated the anticancer activity of alectinib and ceritinib against U87MG, LN229, and GSC23 GBM cell lines. ALK inhibitors effectively induced GBM cell death and inhibited STAT3, causing caspase-dependent/independent cell death when administered alone. Furthermore, antitumor activity was also observed against a temozolomide osimertinib-U87MG cell line. Interestingly, oral administration of alectinib and ceritinib synergistically prolonged the survival of intracerebral GBM xenograft-harboring mice, when compared to controls.

According to Goker Bagca et al. [45] alectinib/TMZ dual treatment exhibited a synergistic reduction of T98G GBM cells viability and decreased expression of PI3K/AKT and their associated genes. Interestingly, while alectinib induces G0/G1 cell cycle arrest, alectinib/TMZ activity promotes S-phase arrest.

### 2.2. AXL Inhibitors

AXL is a transmembrane TK receptor belonging to the Tyro3, Axl, and Mertk (TAM) kinase family and it can be activated by growth arrest-specific protein 6 (GAS6) and Pros1 (Protein S) [46,47]. AXL phosphorylation leads to PI3K and AKT activation. The Gas6/AXL/PI3K/Akt axis protects cells from apoptosis by S6K activation and BCL-2 phosphorylation [44]. AXL is widely expressed in GBM, and its biologically active form Phospho-AXL (P-AXL) is associated with poor prognosis [48]. The receptor activation leads to increased tumor proliferation, cell migration, and angiogenesis in both in vitro and in vivo GBM models [49,50,51].

#### Bemcentinib 

Bemcentinib **4** (Figure 3), also known as R428, is the most specific AXL inhibitor and entered clinical trials in 2014 (i.e., NCT02424617, NCT02922777). It is currently being tested in an early phase I clinical trial on recurrent forms of GBM (NCT03965494) [52,53]. It inhibits AXL with an IC_50_ of 14 nM [50].

Scherschinski et al. [54] tested the combination of bemcentinib and TMZ towards GBM to overcome TMZ resistance and to increase therapy effectiveness. Both naïve (SF126 and U118MG) and TMZ-resistant (SF126-TR and U118MG-TR) cell lines were treated and, whereas single bemcentinib or TMZ administration was ineffective, their combination resulted in a significant reduction of cell survival. Similar results were observed by exposing cells to a bemcentinib/radiation dual treatment vs. the radiation approach [54].

Furthermore, the combinatorial treatment of bemcentinib and the monoclonal antibody Nivolumab prolonged mice survival, suggesting that TKIs can be effective adjuvants to flank traditional therapies, such as PD-1 antibodies [55].

### 2.3. Discoidin Domain Receptor 1 (DDR1) Inhibitors

DDR1 is a receptor TK that is activated by collagens in the extracellular matrix. High levels of collagens have been identified in brain tumors highlighting DDR1 as a valuable target in GBM treatment [56].

#### DDR1 Inhibitors 

Compound **5** (Figure 3), also known as DDR1-IN-1, is a selective and poorly studied DDR1 inhibitor [56], characterized by an IC_50_ of 47 nM [57]. According to Vehlow et al. [56], compound **5** significantly induced both radio-sensitization and chemo-sensitization to TMZ in GBM cell lines.

### 2.4. IGF1R Inhibitors

Insulin-like growth factor 1 receptor (IGF1R) is a ubiquitous receptor TK, activated by both IGF1 and IGF2. It controls several essential cell functions, and is involved in several chronic lung diseases, including cancer. The IGF1R signaling pathway can contribute to neoplastic cells proliferation, migration, survival and chemotherapy resistance. Thus, IGF1R has been evaluated as a pharmacological target for the treatment of several cancers, including NSCLC [58]. Martin et al. [59] observed that in vitro IGF1R nuclear localization increases GBM cell motility and metabolism, while in vivo, IGF1R can translocate to the nucleus, allowing both a higher proliferation rate and the earlier development of GBM.

#### Linsitinib 

Linsitinib **6** (Figure 3), also named OSI-906, is a highly selective and bioavailable IGF1R inhibitor (IC_50_ = 35 nM) [60]. Two phase I clinical trials showed a total response rate of about 30% in advanced solid cancers, and some subjects have obtained enduring advantages from the IGF1R inhibition [61].

Linsitinib slightly decreased GBM cell viability, if compared with a multi-kinase inhibitor named BMS-754807, inducing G1 arrest [62].

### 2.5. MER Inhibitors

Membrane estrogen receptor (MER) TK is a major macrophage receptor associated with apoptotic cells clearance. Its expression is mainly detected in M2 macrophages [63]. Since cancer-associated macrophages often show an M2-like phenotype and participate in cancer progression, angiogenesis, and in tissue remodeling, it seems reasonable to investigate MER as a therapeutic target [63]. MER TK is overexpressed in GBM multiforme, being associated with increased invasiveness. Its depletion changes the glioma cells rounded morphology, also decreasing their invasive potential [64].

The effect of MER on glioma cell invasion is mediated by actomyosin contractility as the expression and phosphorylation of myosin light chain 2 are strongly associated with MER activity. Moreover, the DNA damage increased the upregulation and phosphorylation of MER preventing death cells. This effect is strongly decreased in case of kinase depletion or overexpression of its inactive mutant [64].

#### 2.5.1. MRX2843 

MRX2843 **7** (Figure 3) is a dual inhibitor of MER (IC_50_ = 1.2 nM) and FLT3 and is active against FLT3 point mutations. It also showed therapeutic effectiveness in mouse xenograft models of AC220-resistant acute myeloid leukemia (AML) [65]. Furthermore, RX-2843 synergized with vincristine in the inhibition of acute lymphoblastic leukemia cell lines [66].

Su et al. [67] evaluated the effects of MRX2843 in several GBM cell lines, e.g., GSC407, GSC923, and U251, in macrophages, human brain microvascular endothelial cells (HBMECs), and in a syngeneic GL261 orthotopic GBM murine model. MRX-2843 inhibited cell growth and induced apoptosis in human GBM cells showing after 48 h of treatment EC_50_ values of 95.5, 288.1, and 217.7 nM in U251, GSC923, and GSC407, respectively. A reduction of colony formation was observed after 7–14 days when U251, GSC923, and GSC407 cells were treated with **7** at 100 or 500 nM for only 48 h. It also lowered Mer, AKT and ERK expression, essential for cell survival signaling. It induced an in vivo decrease in both vascular formation and levels of CD206+ glioma-associated microglia and macrophages [67]. The authors showed that MRX-2843 penetrates the blood–brain barrier (BBB) accumulating in the brain tumor tissue with levels about five times higher than the plasma, suggesting a better pharmacokinetic behavior compared to UNC2025 (see next paragraph) [67].

#### 2.5.2. UNC2025

UNC2025 **8** (Figure 3) is an ATP competitive class I MER inhibitor (IC_50_ = 2.6 nM) and showed a favorable pharmacokinetic (PK) profile in mice. Preclinical evaluations in NSCLC models exhibited a 50% reduction in cancer cell survival and colony formation abrogation at a 300 nM dose [68].

Wu et al. [69] studied both in vitro and in vivo UNC2025-induced Mer inhibition. In vitro tests were carried out in several cell lines, i.e., U87, U251, GSC11, GSC20 and EOC2. Among them, GSC11 were the more responsive to proliferation inhibition. Moreover, syngeneic orthotopic allograft mouse GBM models were randomized to receive vehicle (control), UNC2025, fractionated radiation or UNC2025/X-ray combination. Interestingly, while median survival rates were comparable with or without UNC2025, a significant growth delay was observed in UNC2025/X-ray treated mice, with complete responses in 19% of patients. Lastly, UNC2025 lowered CD206+ macrophages levels in mouse cancer models [69].

Analysis of PK profile of UNC2025 in in vivo mouse model of GBM showed a satisfactory BBB permeation and a brain accumulation 4-fold increase with respect to non-tumor brain tissues, demonstrating improved delivery probably due to the tumor microenvironment [69].

### 2.6. c-Met Inhibitors

Mesenchymal-epithelial transition factor (c-MET) is a hepatocyte growth factor (HGF) receptor regulating embryonal cells morphogenesis [70]. It was recently reported to be involved in cancer development regulation [71,72] and several studies have been made to develop c-MET-targeted agents to be employed in several malignancies [73,74]. Interestingly, c-MET expression inhibition was found to block glioma cell growth and migration [75].

#### 2.6.1. Capmatinib

**Figure 4:**
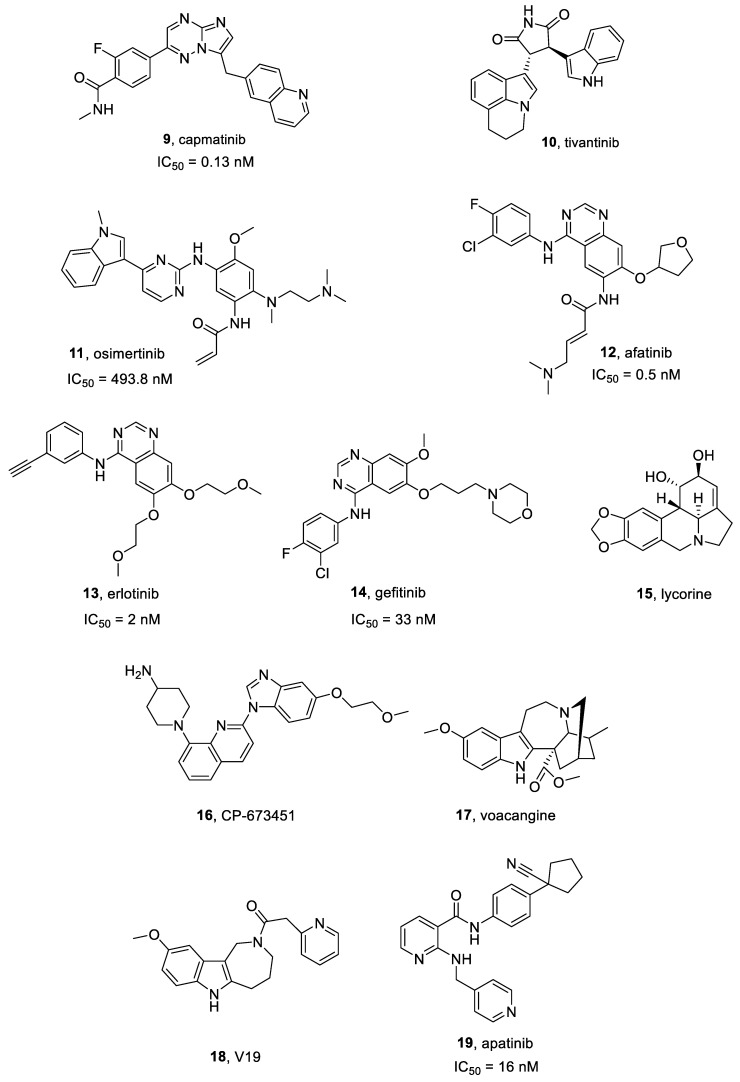
Structures of c-Met, EGFR, PDGFR, and VEGFR inhibitors **9**–**19**.

Capmatinib **9** (Figure 4) is a highly selective c-MET inhibitor (IC_50_ = 0.13 nM [76]), characterized by both in vitro and in vivo effectiveness vs. c-MET-activated preclinical cancer models [77]. It is being investigated as both a single and combinatorial therapeutic agent in several clinical trials [78].

According to Baltschukat et al. [78], capmatinib was highly selective towards c-MET, as well as active against neoplastic models showing c-MET amplification, overexpression, exon 14 skipping mutations or HGF-mediated activation. Interestingly, in vitro sensitivity was observed in autocrine cell lines derived from GBM.

#### 2.6.2. Tivantinib

Tivantinib **10** (Figure 4) is a highly specific small molecule c-MET inhibitor, characterized by antitumor activity in Microphthalmia transcription factor (MITF)-associated tumors [79] and possesses an IC_50_ = 300–400 nM in MKN45, EBC1, and A549 cells [80]. Interestingly, tivantinib also reduced proliferation of multiple myeloma cell lines downregulating c-MET signaling and inhibiting MAPK and PI3K pathways [81].

When tested at high concentration (1 μmol/L) [82], tivantinib inhibited U251 and T98MG GBM cells proliferation and colony formation, while a lower concentration (0.1 μmol/L) did not affect cell proliferation. High concentration of tivantinib in combination with the PI3K inhibitor LY294002 and the mTOR inhibitor rapamycin, strongly inhibited GBM cell proliferation [82].

### 2.7. EGFR Inhibitors

Epidermal growth factor receptor (EGFR) is formed by an extracellular binding domain, a transmembrane portion and intracellular domain endowed with the kinase activity. The receptor is activated by various ligands, including EGF and TGF-α. EGFR activation is induced by both homo- and heterodimerization on the cell surface, leading to the intracellular tyrosine kinase domain phosphorylation [83]. EGFR is overexpressed in both primary (about 60%) and secondary GBMs (about 10%) and is related to the most aggressive GBM forms [83]. Several comparison studies highlighted a significant difference in progression-free survival (PFS) between first and second-generation EGFR inhibitors. For instance, afatinib (second generation) was found to be superior to gefitinib (first generation) in terms of PFS (11 versus 10.9 months) in a phase 2B trial on lung cancer [84]. Third generation inhibitors (i.e., osimertinib) demonstrated a PFS superiority (18.9 versus 10.2 months) compared to second generation ones (i.e., erlotinib and gefitinib) [85].

#### 2.7.1. Osimertinib

Osimertinib **11** (Figure 4) is a selective and irreversible third generation EGFR inhibitor. It is active on EGFR (IC_50_ = 493.8 nM) and different mutated forms, i.e., exon 19 deletion EGFR (IC_50_ = 12.92 nM), and L858R/T790M EGFR (IC_50_ = 11.44 nM) [86]. Osimertinib exhibited significant clinical activity in CNS metastases, due to its optimal permeability through the BBB, higher than other EGFR inhibitors. To date, osimertinib is the golden standard choice for EGFR-mutated NSCLC patients for its efficacy and safety profile [87].

According to Liu et al. [88], osimertinib showed a satisfactory preclinical activity in GBM in both in vitro and in vivo models by inhibiting the growth of six GBM cell lines in a dose-dependent manner. It induced cell cycle arrest, colony formation inhibition, migration and invasiveness of GBM cells. Moreover, osimertinib prolonged animal survival in an orthotopic xenograft murine model.

Osimertinib inhibited EGFRvIII TK with high potency (<100 nM) and its downstream pathway. Moreover, it blocked D317 GSC line growth within in vitro and in vivo models [89].

Analysis of the PK profile of osimertinib showed that the compound well penetrates the BBB in an in vivo mouse model of cancer achieving within three hours after the administration of a single 25 mg/kg oral dose, a brain concentration of 3,695 ± 425 nM, with a brain to plasma ratio >10 [89].

Osimertinib was showed to cause paraptosis in LN-229, U87MG, LN-18 and SF-539 cells. A time-dependent cytoplasmic vacuoles accumulation was observed in such cell lines, while cell membranes and nuclei mostly remained undamaged [90].

#### 2.7.2. Afatinib

**Figure 5:**
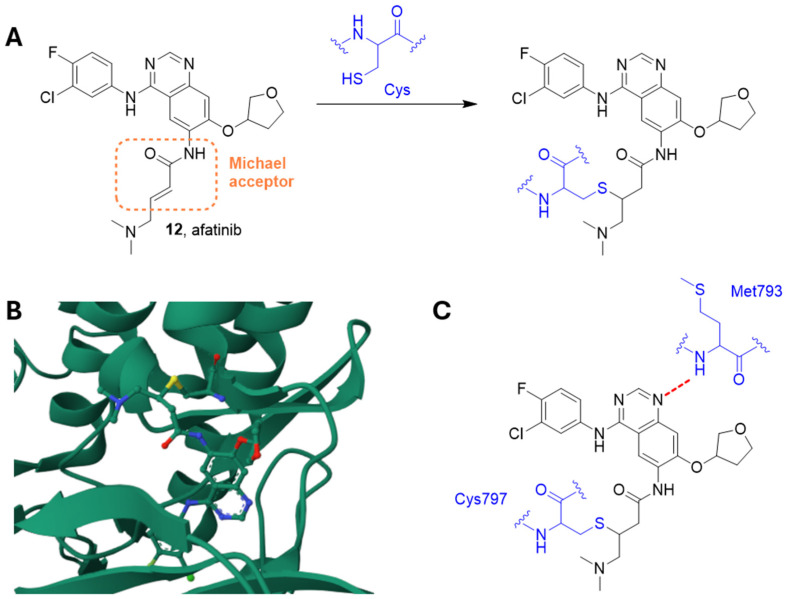
(**A**) The irreversible reaction between afatinib and a residue of cysteine. (**B**) Crystal structure of AGFR in complex with afatinib (PDB: 4G5P). (**C**) Schematic representation of the main interactions between afatinib and EGFR, with amino acid residues crucial to the interaction highlighted in blue. Hydrogen bond is represented as a red dashed line [93].

Afatinib **12** (Figure 4 and Figure 5) is an FDA-approved irreversible EGFR inhibitor (IC_50_ = 0.5 nM) [91], also acting on EGFRvIII mutation [92]. It also irreversibly binds HER2 and HER4.

Indeed, this compound contains an electrophilic group able to give Michael addition with conserved cysteine residues within the catalytic domains of these enzymes, i.e., EGFR Cys797 of EGFR, Cys805 of HER2, and Cys803 of HER4. Analysis of the crystal structure of wild-type EGFR (residues Gly696-Gly1022) in complex with afatinib showed the formation of the irreversible sulfur bridge together with a hydrogen bond between the amide nitrogen of Met793 at the hinge region and the quinazoline core of afatinib (Figure 5) [93].

Recent studies have proven a significant PFS increase in afatinib-treated NSCLC patients with EGFR mutations when compared to gemcitabine plus cisplatin or pemetrexed plus cisplatin treatments. The same parameter was improved in EGFR mutant NSCLC patients with brain metastases [94].

From a PK point of view, afatinib achieves its maximum plasma concentration 2–5 h after oral administration. The contemporary assumption of food significantly reduces the blood levels of the drug. Afatinib possesses a half-life of 37 h and is excreted by feces (about 95%) and urine (about 5%) [91].

Vengoji et al. [92] demonstrated that afatinib and TMZ combination synergistically inhibited proliferation, invasiveness, motility and clonogenic survival of GBM cells, and induced their senescence. Afatinib lowered U87 GBM cells proliferation and motility by inhibiting EGFRvIII and focal adhesion kinase (FAK) signaling pathways, respectively. Noteworthy, afatinib/TMZ association decreased U87 GBM xenograft growth and progression when compared to single drug treatments. These findings encourage afatinib/TMZ use evaluation in EGFR/EGFRvIII GBM patients.

#### 2.7.3. Erlotinib

**Figure 6:**
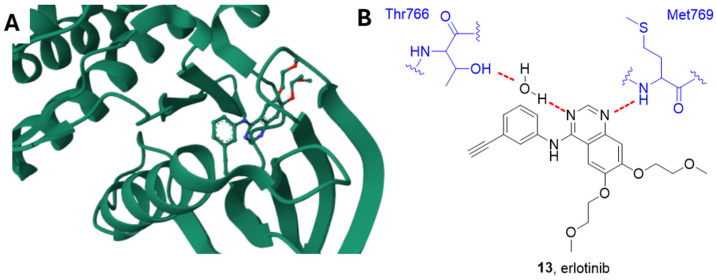
(**A**) Crystal structure of EGFR in complex with erlotinib (PDB: 1M17). (**B**) Schematic representation of the main interactions between erlotinib and EGFR, with amino acid residues crucial to the interaction highlighted in blue. Hydrogen bonds are represented as red dashed lines [96].

Erlotinib **13** (Figure 4 and Figure 6), also known as CP-358774 and OSI-774, is a selective EGFR inhibitor, characterized by an IC_50_ value of 2 nM [95]. It blocks cancer cell proliferation and cell cycle and inhibits downstream EGFR signal transduction.

Erlotinib is an ATP-competitive inhibitor, and the quinazoline scaffold accommodates into the catalytic pocket connecting the N- and C-lobes. According to the study carried out by Stamos et al., the N1 of the quinazoline forms a hydrogen bond with the Met769 amide nitrogen, while the N3 is involved in a water-mediated hydrogen bond with the OH Thr766 side chain (Figure 6) [96].

From a PK point of view, erlotinib showed good oral bioavailability (60% absorption), which can be increased to almost 100% if swallowed with food. Otherwise, the increase in the gastrointestinal pH reduced erlotinib absorption. About drug distribution, more than 90% of erlotinib is bound to albumin and alpha-1 acid glycoprotein. Erlotinib is mainly a CYP3A4 substrate, and about 83% of the administered drug was recovered from feces and 8% from urine, with a half-life of around 36 h [97]. Erlotinib was approved for NSCLC and pancreatic cancer treatments [97].

Amini et al. [98] reported that treating U373 GBM cells with PIK3R3-siRNA plus Erlotinib synergistically induced a decrease in cell survival. Such findings support the hypothesis that PIK3 pathway blockage could heighten the anticancer effects of erlotinib in GBM cells.

A combination of erlotinib and MLN0128 (a mTOR inhibitor) showed a synergistic anticancer activity inhibiting p-EGFR, MAPK and PI3K pathways in vitro. This combinatorial treatment also inhibited tumor growth in an orthotopic xenograft murine model, significantly prolonging GBM-bearing mice survival [99].

Moreover, it was demonstrated that treating U251MG cells with both erlotinib and the alkaloid oxymatrine significantly lowered p-EGFR, p-Akt and p-mTOR levels, and inhibited cancer cell proliferation, compared to erlotinib or oxymatrine monotherapies [100].

Mesbahi et al. [101] evaluated the erlotinib/arsenic trioxide combination on both U87-MG and A172 GBM cells. Their results showed a reduction of cell proliferation and metabolic activity. Interestingly, such combination induced G2/M cell cycle arrest, as well as an apoptotic cell death rate and significant increase in ROS (reactive oxygen species). These results suggest that EGFR inhibition could overcome GBM resistance to arsenic trioxide treatment.

#### 2.7.4. Gefitinib

Gefitinib **14** (Figure 4), also known as ZD1839, is a selective ATP-competitive EGFR and HER-2 inhibitor, which displays an IC_50_ of 33 and 79 nM towards EGFR and HER-2, respectively [102,103]. It is the first EGFR-targeting anticancer molecule lunched in Japan, Australia and USA for NSCLC treatment. Gefitinib is prescribed as a monotherapy to treat both locally advanced or metastatic NSCLC after failure of docetaxel and platinum-based chemotherapies. In preclinical studies, gefitinib showed anticancer effects against several human cancer cell lines expressing EGFR (e.g., lung, ovarian, breast and colon cancers). In GBM murine xenograft models, gefitinib combined with others cytotoxic agents produced both cancer regression and tumor growth inhibition, improving the rate of survival. Gefitinib in combination with temozolomide in U87MG cell line induced cytotoxic effects with IC_50_ values of 11 μM [104].

#### 2.7.5. Lycorine

Lycorine **15** (Figure 4) is a pyrrolo[3,2,1-*de*]phenanthridine ring-type alkaloid extracted from different Amaryllidaceae genera, and is characterized by several biological properties, such as anti-cancer, antiviral and anti-inflammatory effects. Lycorine exhibited cytotoxic effects on various malignancies, e.g., cervical cancer, leukemia, multiple myeloma, hepatocellular carcinoma, prostate cancer, bladder cancer, breast cancer and GBM [105].

Lycorine lowered U251 GBM cells proliferation, colony formation and migration by causing EGFR-mediated apoptosis. Moreover, it inhibited the cancer growth in different in vivo murine models, e.g., a U251-luc intracranially orthotopic xenograft, an EGFR knockdown U251 subcutaneous xenograft, and a patient-derived xenograft model [105].

### 2.8. PDGFR Inhibitors

Platelet-derived growth factor receptors (PDGFRs) are transmembrane receptor TKs. Their signaling is mediated by monomeric PDGFRs dimerization and intracellular tyrosine autophosphorylation. PDGFR pathway is crucial for cancer growth, angiogenesis and lymphangiogenesis. Aberrant PDGFR signaling has been observed in several malignancies, i.e., glial brain tumors, chronic myelomonocytic leukemia, prostate, lung/breast adenocarcinoma, HCC and NSCLC [106]. PDGFR and its ligand PDGF are co-expressed in GBM. Interestingly, PDGFR mutations or amplification have been identified as indicators of GBM subgroups originating from a PDGF receptor-responsive cell [107].

#### CP-673451

CP-673451 **16** (Figure 4) is a potent PDGFR inhibitor, about 450-times more selective for PDGFRβ (IC_50_ = 1 nM in cells) than vs. other PDGFR subclasses [108,109].

Lane et al. [105] identified CP-673451 as an inducer of neurite-like outgrowth in three GBM cell lines (U87, U251 and LN229) and astrocytes, suggesting that the compound promotes cell differentiation into neural-like cells with a positive effect on GBM patients. Furthermore, CP-673451 improved anticancer TMZ activity in a U87 xenograft GBM murine model. Cancer volumes were significantly reduced by the combination treatment respect to both singular treatments [110].

The analysis of the PK profile of CP-673451 in a GBM rat model showed that a single oral dose of 50 mg/kg determined a plasma concentration above the EC_50_ for ~4 h [109].

### 2.9. VEGFR Inhibitors

Vascular endothelial growth factors receptors (VEGFRs) regulate both vasculogenesis and angiogenesis [111]. VEGFR family includes three members, i.e., VEGFR-1, VEGFR-2 and VEGFR-3. VEGFR-1 and VEGFR-2 play crucial roles in both physiological and pathological angiogenesis, including cancer angiogenesis. VEGFR-3 is involved in angiogenesis regulation in early embryogenesis, as well as functioning as lymphangiogenesis critical regulators [111]. In GBM, VEGFRs are highly upregulated, and the expression degree was associated with the malignancy grade. VEGFR signaling plays a key role in the development of the GBM immunosuppressive tumor microenvironment [112].

#### 2.9.1. VGB

VGB is a peptide (sequence 2HNCIKPHQGQHICNDE-COOH) [113], specifically developed to bind both VEGFR1 and VEGFR2. Inhibitor design was based on residues contained in VEGFA, involved in the interaction with VEGFR1 and VEGFR2.

This peptide inhibits U87 GBM cells proliferation with a IC_50_ of 0.92 μM. Since, U87 GBM cells over-expressed VEGFR2, these data confirm the involvement of this receptor subtype in GBM [113].

#### 2.9.2. Voacangine

Voacangine **17** (Figure 4) is an indole alkaloid isolated from *Voacanga Africana* and *Tabernaemontana Catharinensis* root barks [114]. It significantly suppresses VEGF-induced tube formation and chemoinvasive angiogenetic processes in vitro models. Furthermore, voacangine inhibits in vivo angiogenesis in chorioallantoic membrane at non-toxic concentrations. Moreover, decreased hypoxia inducible factor-1a and its target gene (VEGF) expression levels, in a dose-dependent manner [114].

Cho et al. [115] developed a set of voacangine analogues, specifically targeting and modulating VEGFR2 activity. Among these compounds, V19 **18** (Figure 4) increased antiangiogenic activity against VEGF-induced VEGFR2 phosphorylation in a U87 GBM mouse xenograft model, without cytotoxic effects [115].

#### 2.9.3. Apatinib

Apatinib **19** (Figure 4), also named Rivoceranib, is a small molecule TKI, approved in China for gastric cancer treatment. It was showed to block tumor angiogenesis by inhibiting VEGF signal transduction, particularly, binding VEGFR2 (IC_50_ = 16 nM) [116].

When tested in 15 patients (after chemoradiotherapy) suffering from recurrent grade IV GBM, apatinib exhibited a PFS of 2 months, as well as a median overall survival rate of 6.5 months with the most common side effects being thrombocytopenia, asthenia and hand–foot syndrome [117].

### 2.10. FAK Inhibitors

Focal adhesion kinase (FAK) is a non-receptor TK, which acts as an adaptor protein regulating adhesion signaling and cell migration. It can also promote cell survival responding to stress stimuli. FAK transduces signals ranging from cell adhesions to regulation of functions altered in cancer, like cell survival, migration, and invasion [118].

#### PF573228

**Figure 7:**
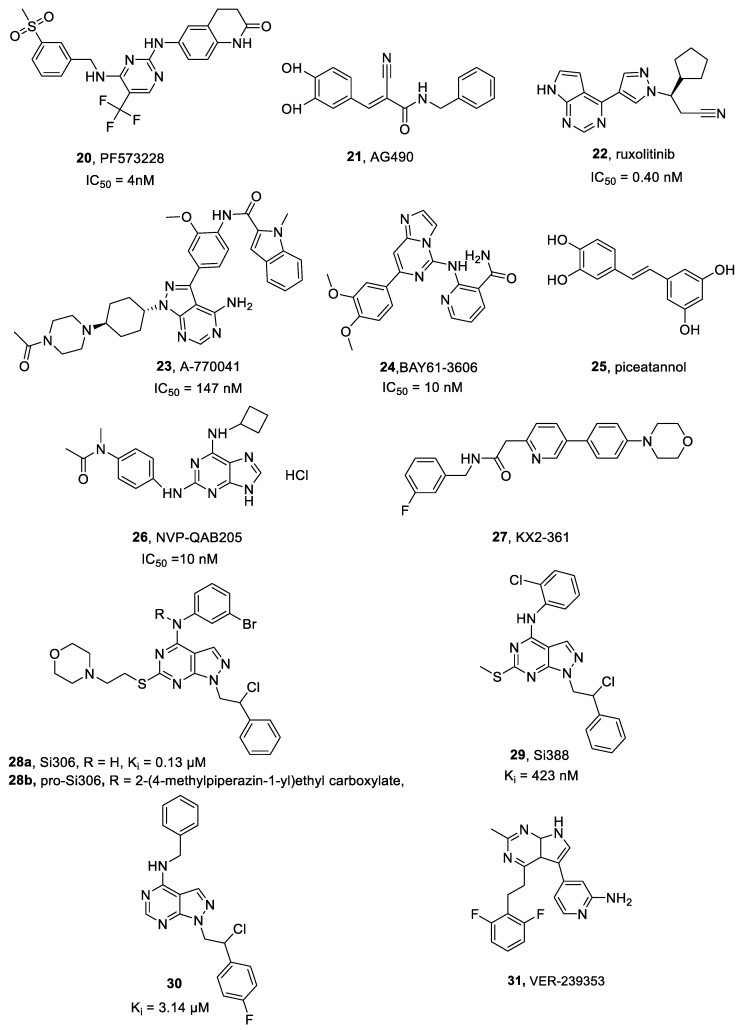
Structures of FAK, JAK, LCK, SYK, Src, and DYRK1A inhibitors **20**–**31**.

PF573228 **20** (Figure 7) is a small molecule inhibitor, selectively targeting FAK’s ATP-binding site (IC_50_ = 4 nM) [119] and effectively blocking its catalytic activity in several cell lines including neuroblastoma cells [120].

Nguemgo Kouam et al. [121] showed that PF573228 reduced the adhesion of U87 and U373 GBM cells. Interestingly, another study [122] showed that treating GBM cells with PF-573228 arrested proliferation and decreased cell size. It also lowered GBM neurosphere growth.

### 2.11. JAK Inhibitors

Janus kinases (JAKs) are non-receptor TKs that include four members, i.e., JAK1, JAK2, JAK3 and TYK2 [123]. JAK/STAT pathway is activated by many protein ligands including cytokines, growth factors, peptide hormones and interferons, and regulates several cellular processes like cell proliferation, growth, differentiation, and apoptosis. Persistent or dysregulated JAK/STAT3 signaling is involved in many diseases characterized by chronic inflammation and fibrosis, as well as cancer [124]. In GBM, the inhibition of JAK/STAT pathway is involved in the regulation of inflammatory response, frequently present in malignancies [125].

#### 2.11.1. AG490

AG490 **21** (Figure 7) is a TKI specific for JAK2, able to decrease STAT3 phosphorylation. Its therapeutic potential has been proved in brain hemorrhage, fibrosis and liver injury treatments [126].

Lebedev et al. [127] evaluated AG490 in neuroblastoma, GBM, breast and NSCLC cells. GBM cells resulted to be sensitive to the compound and the effect was increased when used in combination with doxorubicin.

#### 2.11.2. Ruxolitinib

Ruxolitinib **22** (Figure 7) is a reversible class I JAK inhibitor that competes with ATP in the JAK kinase catalytic site (IC_50_ = 0.40 nM) and was approved for myelofibrosis treatment. Its effectiveness in myelofibrosis has been mainly associated to the reduction of the inflammation induced by constitutive JAK-STAT activation, as well as by a non-specific myelosuppression [128,129].

From a PK point of view, ruxolitinib achieves peak plasma concentrations within one hour after administration and possesses a half-life of 2.3 h [128].

Delen and Doganlar [130] showed that ruxolitinib inhibited JAK/STAT pathway in GBM spheroids in a dose-dependent manner.

Ruxolitinib was demonstrated to improve TMZ’s apoptotic effects on U87MG cells, BCSCs (brain cancer stem cells) and HBMECs (human brain microvascular endothelial cells). Interestingly, it regulates the WNT signaling pathway both as single agent and in combination with TMZ [131].

### 2.12. LCK Inhibitors

Lymphocyte-specific protein tyrosine kinase (LCK) is a non-receptor TK, member of the Src family. It is involved in T-cell receptor signaling, also playing a crucial role in mediating B-cell receptor signaling in chronic lymphocytic leukemia cells. Furthermore, its expression was detected in several neural tissues, including hippocampus, cerebellum, and retina [132].

LCK was expressed at a high level in primary central nervous system lymphoma patients but at a low level in GBM patients. However, LCK expression positively correlated with the levels of infiltrating B cells in diffuse large B-cell lymphoma and GBM [132].

#### LCK-I

A-770041 **23** (Figure 7) is a highly selective small molecule LCK inhibitor (IC_50_ = 147 nM) [133].

According to Zepecki et al. [134], A-770041 mediated Lck inhibition and Crk-II phosphorylation, pseudopodia formation and migration of human glioma stem cells (hGSCs). A-770041 in vivo intraventricular administration, employing an orthotopic xenograft glioma model, significantly decreased the cancer size. Moreover, treating of hGSCs with A-770041 resulted in a significant inhibition of self-renewal and cancer-sphere formation.

### 2.13. SYK Inhibitors

Spleen tyrosine kinase (SYK) is a cytoplasmic enzyme involved in mediating antigen-associated signals in both innate and adaptive immune systems [135]. Its activity is necessary to B cells, mast cells and for platelet propagation/activation. After being recruited, SYK can undergo to autophosphorylation, as well as to phosphorylation by Src kinase. Upon activation, SYK triggers multiple signaling cascades, giving several inflammatory or immunological outcomes. Thus, SYK could be pharmacologically targeted to modulate and treat inflammatory and autoimmune diseases, as well as hematological cancers [130]. SYK was expressed in both human and murine glioma cell lines. Its inhibition blocked migration, proliferation and colony formation [136].

#### Bay61-3606, Piceatannol and NVP-QAB205

BAY61-3606 **24** (Figure 7) is a selective SYK inhibitor (IC_50_ = 10 nM [137]), characterized by anti-inflammatory effects on pathological processes like acute kidney injury. It exerts its activity by suppressing inflammatory macrophage response [138].

Piceatannol **25** (Figure 7), otherwise called Pic, a resveratrol derivative, is a selective SYK micromolar inhibitor [139]. It inhibits phosphorylated SYK expression and suppressed both migration and invasiveness of CAL27 (tongue squamous carcinoma) cells in a wound healing assay evaluation [138].

NVP-QAB205 **26** (Figure 7) is an effective SYK phosphorylation inhibitor (IC_50_ = 10 nM [140]) and demonstrated excellent activity in preventing human mast cell and basophil activation [141].

Moncayo et al. [136] tested the above cited small molecule inhibitors on four different cell lines: BS287 GBM-derived spheres, U87, SF767 and BS125 GBM cells showing cell proliferation inhibition in all lineages. Piceatannol was the best proliferation inhibitor for both BS287 and U87 cells, while BAY61-3606 reached the most satisfactory results on SF767 and BS125 cell lines, followed by NVP-QAB205. Compound **24** decreased in vivo GBM growth and invasiveness, also reducing B and CD11b+ cell mobility and infiltration [136].

### 2.14. Src Inhibitors

Src family is a class of non-receptor TKs, playing a pivotal role in several cancers [142]. SRC activity is involved in GBM through the regulation of networks that control inflammation and metabolism [143,144].

#### 2.14.1. KX2-361

KX2-361 **27** (Figure 7) is a novel, non-ATP competitive small molecule Src inhibitor with a satisfactory cytotoxic activity against several CNS cancer cell lines [145].

KX2-361 was investigated against human and murine glioma cells and also in a syngeneic orthotopic GBM murine model [146]. Interestingly, it reduced Src autophosphorylation, and disrupted microtubule structure in the GL261 murine GBM cell line.

#### 2.14.2. Si306 and Analogue Compounds

**Figure 8:**
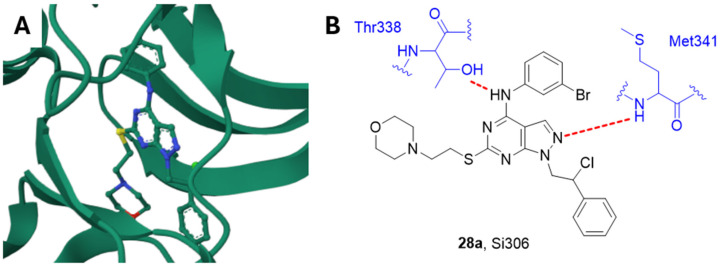
(**A**) Crystal structure of Src in complex with Si306 (PDB: 4O2P). (**B**) Schematic representation of the main interactions between Si306 and Src, with amino acid residues crucial to the interaction highlighted in blue. Hydrogen bonds are represented as red dashed lines [147].

In recent years, we obtained promising results in developing compounds active against GBM by inhibiting Src. Derivative Si306 **28a** (Figure 7 and Figure 8) is an ATP-competitive small molecule Src inhibitor (K_i_ = 0.13 μM) [147,148] which accommodates into the Src catalytic pocket forming two hydrogen bonds, one between the C4 amino group and the Thr338 OH side chain, and the other one between the N2 of the pyrazolopyrimidine and the NH of Met341 (Figure 8) [147].

Furthermore, SI306 is able to induce apoptosis in patients derived invasive GBM cell lines [149] and in combination with radiotherapy significantly inhibited U87 GBM xenografts growth in nude mice, compared to both control and single treatment [150]. In an in vitro assay performed on human GBM cell lines (i.e., U87 and multidrug resistant U87-TxR cell lines), and three primary GBM cell cultures, Si306 and its prodrug (pro-Si306) **28b** (Figure 7) showed a considerably degradation of the extracellular matrix emerging as potential GBM aggressiveness suppressors. In vivo, the two compounds exhibited an anti-invasive effect against U87 xenografts in zebrafish embryos [151]. The in vitro and in vivo PK profiles of Si306 and pro-Si306 were determined. Both molecules showed good metabolic stability, pro-SI306 showing an increasing water solubility compared to the parent compound. Furthermore, the prodrug showed comparable efficacy and a slightly increased median survival time of mice in an orthotopic GBM model with respect to Si306 [150]. Moreover, Si306 and its prodrug exhibited high pro-oxidative potential in patient-derived GBM cells determining an increase in ROS synthesis followed by double-strand DNA breaks, mitochondrial membrane potential disruption and senescence [152].

Lastly, Si306 and pro-Si306 autophagy-inducing ability was evaluated in both U87 and U87-TxR cells. Si306 and pro-Si306 significantly inhibited cell proliferation and triggered necrosis when administered in combination with the autophagy inhibitor bafilomycin A1 [153].

Compound Si388 **29** (Figure 7) is another pyrazolo-pyrimidine Src inhibitor (K_i_ = 423 nM). It affected tumorigenicity and cell viability in 2D and 3D GBM cellular models (T98G, U251 and GBMSC83 cell lines), and it enhanced cancer cell sensitivity to ionizing radiation [154].

Compound **30** (Figure 7) showed a promising inhibitory activity against Src (K_i_ = 3.14 μM) and Abl (K_i_ = 0.44 μM) kinases, cell viability reduction towards U-87, LN-18, LN-229 and DBTRG GBM cell lines. Compound **30** in vitro ADME (absorption, distribution, metabolism and excretion) evaluation showed high metabolic stability and a satisfactory passive permeability across gastrointestinal and blood–brain barriers [155].

#### 2.14.3. TAT-Cx43

TAT-Cx43 peptide (sequence TAT-AYFNGCSSPTAPLSPMSP) inhibits Src oncogenic activity, especially in GSCs, and decreases their survival, invasiveness and tumorigenicity. Furthermore, it boosts survival rate in animal models [156].

Furthermore, TAT-Cx43 decreased glucose uptake in human GSCs and reduced oxidative phosphorylation without a compensatory increase in glycolysis [157].

### 2.15. Dual-Specificity Tyrosine Phosphorylation-Regulated Kinase 1A (DYRK1A) Inhibitors

DYRKs belong to a group of proline-directed kinases and include five members, DYRK1A, DYRK1B, DYRK2, DYRK3 and DYRK4 [158]. Among them, DYRK1A is involved in GBM cell cycle arrest [159], as well as in promotion of cyclin B degradation in GSC cells, decreasing cyclin dependent kinase (CDK) activity and inducing cell cycle arrest [160].

#### VER-239353

VER-239353 **31** (Figure 7) is a potent and selective DYRK1A/1B inhibitor (IC_50_ = 7 and 2.4 nM, respectively) with a partial activity towards DYRK2 (>30-fold selectivity) [161]. DYRK1A/B inhibition by VER-239353 in U87MG cells increased retinoblastoma protein (pRb) and cyclin D1, as well as cell cycle inhibitors p21 and p27. This led to an exit from G0 phase and a following arrest in G1. VER-239353 also reduced U87MG cell proliferation in both 2D and 3D culture. In vivo, VER-239353 induced proliferation stasis in a U87MG xenograft cancer model employing female nude mice [161].

### 2.16. MEK Inhibitors

Mitogen-activated protein kinases (MEKs) are a class of enzymes which can phosphorylate both tyrosine and serine/threonine residues. They are involved in human cancers through the activation of Ras/Raf/MEK/ERK transduction cascade [162]. In GBM, MEK/ERK pathway has been showed to enhance both cell migration and invasion [163].

#### 2.16.1. Binimetinib

**Figure 9:**
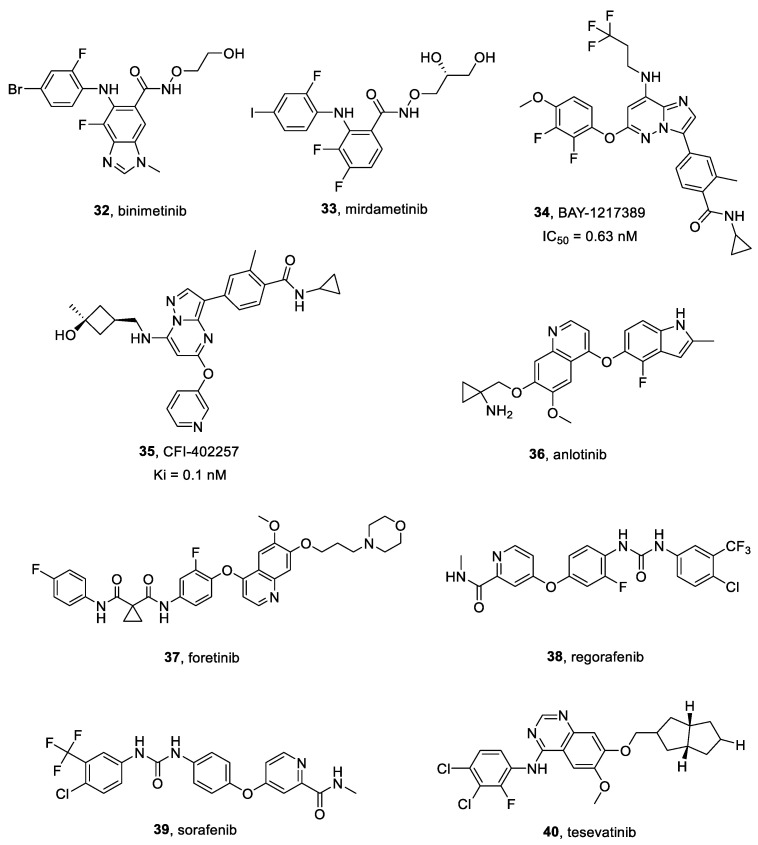
Structures of MEK, TTK, and multitargeted inhibitors **32**–**40**.

Binimetinib **32** (Figure 9) is a selective and allosteric inhibitor of MEK1/2. It was approved in several countries in combination with the selective B-RAF protein inhibitor encorafenib for patients with unresectable or metastatic melanoma. Binimetinib decreased pERK levels when tested in multiple cancer cell lines, ingluding neuroblastoma [164,165].

In vitro studies were performed by Bikhezar et al. [166], employing multicellular U87 human GBM spheroids. Binimetinib loaded nanocarriers inhibited spheroids growth, showing a synergistic effect when administered in combination with radiation both in vitro and in vivo (GBM murine xenografts) [167]. Furthermore, an additive effect was observed when it was combined with TMZ [167].

#### 2.16.2. Mirdametinib

Mirdametinib **33** (Figure 9), also known as PD-0325901, is a selective MEK1/MEK2 inhibitor, characterized by an effective blood-brain barrier permeability, when compared to other MEK inhibitors. Clinical trial NCT04923126 (still in recruiting phase) was launched to evaluate its safety, preliminary efficacy and PK in patients suffering from pediatric low-grade glioma (pLGG) [168].

According to Houweling et al. [169], mirdametinib showed an in vitro radio sensitizing effect on GBM8 spheroids. Mice treated with radiation alone or combined with mirdametinib exhibited significantly better survival if compared to control.

### 2.17. TTK Inhibitors

Threonine tyrosine kinase (TTK), otherwise called MPS1 (monopolar spindle 1) kinase, is a multi-specific enzyme that phosphorylates tyrosine, serine and threonine residues [170]. TTK is essential for in vitro clonogenicity and in vivo tumor propagation in GSCs. TTK expression is high in GBM and is correlated with a poor prognosis in GBM patients [171].

#### BAY-1217389 and CFI-402257

BAY-1217389 **34** (Figure 9) is a selective TTK inhibitor, competitively binding its ATP site (IC_50_ = 0.63 nM) [172]. It is characterized by a favorable PK profile, a high distribution volume and low blood clearance [173].

CFI-402257 **35** (Figure 9) is a highly selective small molecule TTK inhibitor (Ki = 0.1 nM [174]), currently employed in a phase two clinical trial testing its safety and tolerability in breast cancer patients (NCT02792465). It can suppress HCC cell proliferation in a dose-dependent manner, as well as induce cell apoptosis [175].

Different concentrations of BAY-1217389 and CFI-402257 showed a significant growth suppression effect on both U87 and U251 GBM cells in a dose dependent manner [173]. BAY-1217389 and CFI-402257 significantly inhibited GBM cells colony formation. BAY-1217389 and CFI-402257 in combination with TMZ showed a higher cell viability decrease in both U251 and U87 cells than TMZ alone [173].

### 2.18. Multitarget Inhibitors

Targeting more than one kinase can lead to an effective increase due to a synergistic action and a reduction of the drug resistance. Several single kinase inhibitors resulted to be multi-target inhibitors due to the kinase ATP-binding sites homology. Moreover, the optimization of potent single kinases or the combination of selective agents led to development of many multi-target inhibitors [176].

#### 2.18.1. Anlotinib

Anlotinib **36** (Figure 9) is small-molecule multitarget TKI, specific for VEGFRs, Kit, PDGFR-α and FGFRs [177]. Its IC_50_ towards VEGFR2 is 0.2 nM and it inhibited FGFR1 of 45.0% at 1 μM [177,178]. Moreover, it inhibited cancer angiogenesis, cell proliferation, cell migration, VEGFR-induced HUVEC cell proliferation, and VEGF/PDGF-BB/FGF-2-induced formation of capillary-like tubes in endothelial cell cultures. Furthermore, anlotinib suppressed HUVEC migration, tube formation and microvessel growth in vitro, and reduced vascular density in vivo.

Xu et al. [179] reported that anlotinib lowered the proliferation, migration and invasiveness of GBM cells (A172, U87, U251) in a dose-dependent manner. Moreover, anlotinib anti-GBM activity was increased by TMZ [179].

Evaluation of the PK profile in several solid tumors highlighted that anlotinib is quickly absorbed through the intestine with comparable brain and plasma distribution [180].

#### 2.18.2. CR13626

CR13626 (chemical structure was not disclosed by Rottapharm Biotech company), a brand-new brain penetrant small molecule multitarget TKI, inhibited the in vitro activity of several kinases involved in GBM development, e.g., EGFR (IC_50_ = 3 µM) [174], VEGFR2, Fyn, Lck, Yes, RET and HGK.

Galimberti et al. evaluated in vivo CR13626 administration. Oral treatment in an orthotopic murine GBM xenograft model resulted in a time-dependent reduction of cancer growth, causing a significant increase in animal survival [181].

#### 2.18.3. Foretinib

Foretinib **37** (Figure 9) is a multi-kinase inhibitor developed as an ATP-binding site competitor. Foretinib is an oral multikinase inhibitor targeting c-MET, RON, Axl, Tie-2, VEGFR, c-KIT, Flt-3, and PDGFR signaling pathways. As a potent c-MET inhibitor, foretinib acted on several c-MET-activated cell lines, reducing cancer growth in different animal models. Moreover, it inhibited the TAM family of RTKs, killing GBM cells [182].

Gortany et al. [183], reported that foretinib inhibited GBM cell proliferation through a G2/M cell cycle arrest and mitochondrial-mediated apoptosis. Moreover, foretinib lowered GBM cells invasiveness by downregulating MMP2, uPA and uPAR proteolytic cascade and epithelial-mesenchymal transition (EMT)-related genes [183].

#### 2.18.4. Regorafenib

Regorafenib **38** (Figure 9) is a small molecule multi-target TKI active against VEGFR-1, VEGFR-2, VEGFR-3 with IC_50_ values of 13 nM, 4.2 nM and 46 nM, respectively [184]. It also inhibited the mutant oncogenic kinases KIT, RET and B-RAF, suppressing both cancer angiogenesis and cell proliferation.

Chiang et al. [185] observed that regorafenib significantly increased TMZ-induced apoptosis, also suppressing both invasiveness and migration potential in U87 and GBM8401 cells. Orthotopic xenograft experiments showed tumor size reduction, as well as prolonged survival in combinatorial administration, even with TMZ half-dose [185].

Regorafenib is a CYP3A4 substrate and is primarily metabolized in the liver to the corresponding N-oxide and demethylated N-oxide metabolites, which show similar kinase inhibitory activities. Although levels of regorafenib and its metabolites are lower in the cerebrospinal fluid than in plasma, it seems reasonable that sufficient CNS concentrations of these compounds are temporarily reached in glioma patients [186].

#### 2.18.5. Sorafenib

Sorafenib **39** (Figure 9) is a potent multi-target kinase inhibitor, that suppressed cancer cells proliferation by inhibiting the activity of RAF-1, B-RAF and RAS/RAF/MEK/ERK signaling pathways [187]. It also inhibited angiogenesis through targeting c-Kit, FLT-3, PDGFR-β, VEGFR-2, VEGFR-3 and other TKs.

Kim et al. [188] confirmed the combinatorial effectiveness of sorafenib plus TTFields, in GBM. Sorafenib prevented cancer expansion in murine GBM xenografts decreasing STAT3 levels and increasing sensitivity towards TTFields. Furthermore, sorafenib plus TTFields significantly inhibited tumor growth. Combinatorial treatment more effectively reduced STAT3 expression in vivo than in U87 and U373 cells [188].

Zajak et al. performed a study aimed at evaluating the synergistic effects [181] of Sorafenib plus LY294002 in T98G cells. Simultaneous treatment with both compounds was more effective in inducing apoptosis than single applications. Effectiveness was associated with both mitochondrial membrane potential decreasing and Cas3/9 activation. Raf and PI3K expression was also inhibited [189].

#### 2.18.6. Tesevatinib

Tesevatinib **40** (Figure 9) is an effective multi-TKI (IC_50_ = 0.3 nM for EGFR), employed in a phase II clinical trial for autosomal dominant polycystic kidney disease treatment (NCT01559363). Tesevatinib exhibited a satisfactory inhibition profile for Src kinase, also decreasing activity of both EGFR and cAMP pathways [190].

Kizilbash et al. [191] evaluated tesevatinib in vitro cytotoxicity on patient derived GBM12 and GBM6 cells. Tesevatinib efficiently reduced cell viability and inhibited EGFR signaling in vitro. They also carried out in vivo experiments by employing murine models bearing either intracranial or flank GBMs. However, its effectiveness, when compared to vehicle in intracranial and flank GBM models, was found to be modest and due to partial EGFR signaling inhibition [191].

The same authors showed that tesevatinib efficacy in EGFR-amplified patient-derived xenograft GBM models is higher in vitro than in vivo, despite a significant distribution in the brain with respect to the plasma, probably because of drug-tissue binding and compensatory signaling [191].

## 3. Challenges and Opportunities for Drug Delivery of Small Molecules Acting as Tyrosine Kinase Inhibitors

The effectiveness of chemotherapy drugs for the treatment of malignant brain tumors is mainly limited by the BBB presence. Thus, the drug efficacy depends on the capacity to cross adequately the BBB reaching a therapeutic level. The main failure of TKIs in the treatment of GBM is due to their poor BBB permeability and the lack of tumor specificity [192]. Although BBB may be chemically or physically disrupted to enhance permeability through methods such as bradykinin analogues-induced tight junction alterations, osmotic disruption using mannitol and focused ultrasound techniques [193], several drug delivery strategies are being studied to overcome the weakness of TKIs and improve their BBB permeability and tumor specificity [192].

Nanocarriers, such as liposomes, polymeric nanoparticles/micelles, albumin nanoparticles, inorganic nanocarriers and lipid nanocapsules, are currently studied to better reach GBM brain sites [192].

Liposomes, composed of concentric single or multiple lipid bilayers with an aqueous core, can contain both hydrophilic and lipophilic molecules. They are morphologically like cellular membranes, biocompatible and non-immunogenic [194]. Such a technology has been employed to deliver doxorubicin and erlotinib, using transferrin to target transferrin receptors overexpressed on brain endothelial and GBM cells, as well as a cell-penetrating peptide to enhance intracellular uptake of carriers [195].

Polymeric nanoparticles, used to specifically deliver drugs to various malignancies, can be characterized by several compositions of natural polymer nanomaterials (e.g., cellulose, chitosan, gelatine, alginate and hyaluronic acid) or synthetic polymers (e.g., polycaprolactone, polyvinyl alcohol, poly-lactide-co-glycolic acid, polyethyleneimine, and polylactic acid) [196]. For instance, optimized imatinib mesylate loaded poly-lactide-co-glycolic acid nanoparticles were functionalized with Pluronic^®^ P84, to overcome the drug P-glycoprotein efflux mediated that is increased in GBM cells [197].

Micelles are formed via amphiphilic block copolymers self-associating in aqueous solution [198]. They are largely employed as drug carriers due to their properties such as narrow size distribution, thermodynamic stability, and suitability to carry hydrophobic molecules [198,199]. Interestingly, Greish et al. [200] encapsulated both dasatinib and crizotinib in poly (styrene-co-maleic acid) micelles affording a more selective distribution and a reduced systemic toxicity in GBM.

Albumin is a plasma protein characterized by both a great safety profile and enhanced permeability and retention effect-induced high accumulation in solid cancers. Such a set of features makes albumin an ideal tool to develop drug delivery systems for malignancies targeting [201]. Yang et al. [202] formulated human albumin-based nanoparticles to deliver both ibrutinib and hydroxychloroquine in a glioma animal model, demonstrating nanoparticles accumulation at the tumor site after intravenous injection.

Inorganic nanocarriers drug delivery vehicles, such as gold, silica, graphene, and carbon nanotubes have been employed due to their versatile physicochemical properties (i.e., easy availability/functionalization, accumulation in cancer cells without recognition by P-glycoprotein) [203]. Moore et al. [204] used carbon nanotubes with multiple polymer coatings to enhance both the therapeutic efficacy and the release kinetics of dasatinib, although carrier toxicity is still questioned.

Lastly, lipid nanocapsules are being studied as drug carriers due to their many advantages, such as high stability, high drug loading, and the opportunity for easy production scale-up [205]. For instance, Clavreul et al. [206] overcame the limitations of free drug in GBM treatment loading sorafenib into these nanocapsules.

## 4. Conclusions

GBM is both the most aggressive and common type of malignancy originating in the brain. It is characterized by a very poor prognosis, with a median value at diagnosis of 65-years-old, and a male sexual predisposition. Many kinase inhibitors are being tested for the treatment of GBM but none of these compounds have been approved, alone or in combination to treat this tumor. Several inhibitors are currently in clinical trials, as reported in Table 1. Although numerous compounds have demonstrated noteworthy in vivo efficacy, the heterogeneity of the disease supports the employment of combinatorial therapeutic regimens. In particular, the complexity of glioblastoma biology, coupled with the onset of resistance mechanisms, underscores the urgency to develop personalized approaches. The combination of immunotherapy with selected TKIs, guided by patient categorization based on specific kinase overexpression, could unlock the full therapeutic capabilities of TKIs and reshape the glioblastoma treatment landscape.

Furthermore, the need for compounds to cross the BBB emphasizes the demand for innovative TKI delivery strategies. In this context, nanotechnology and targeted drug delivery systems emerge as promising approaches for optimizing the pharmacokinetics and biodistribution of TKIs in GBM therapy with good chances of translating these findings into impactful clinical applications.

## Figures and Tables

**Table 1:** Clinical Trials involving tyrosine kinase inhibitors.

Compound	Drug Combined with	Pharmaceutical Company	Targeted Kinase	NCT Number	Phase	Status
Adavosertib [207]	-	-	WEE1	NCT02207010	0/1	Completed
Axitinib [208]	Lomustine	Pfizer	VEGFR	NCT01562197	2	Completed
Bevacizumab [209]	Buparlisib	Novartis	VEGFR, Src	NCT01349660	1/2	Completed, has results
Ceritinib [210]	-	Novartis	ALK, IGFR1, FAK	NCT02605746	0/1	Completed
Entrectinib [211]	-	Hoffmann-La Roche	TRK, ALK, ROS1	NCT02650401	1/2	Ongoing
Ponatinib [212]	-	-	VEGFR, PDGFR, FGFR, Src	NCT02478164	2	Completed, has results
Regorafenib [213]	Lomustine	Bayer	VEGFR, TIE-2, PDGFR, FGFR, KIT, RET, RAF	NCT02926222	2	Completed
Dasatinib [214]	Bevacizumab	-	VEGFR, Src	NCT00892177	1/2	Completed, has results
Dasatinib [215]	Crizotinib	Pfizer	Src, c-MET	NCT01744652	1	Completed
Epitinib [216]	-	-	EGFR	NCT03231501	1	Completed
Anlotinib [217]	-	-	VEGFR, FGFR, PDGFR, c-Kit	NCT04004975	1/2	Completed
Anlotinib [218]	Temozolomide	-	VEGFR, FGFR, PDGFR, c-Kit	NCT04547855	2	Completed
Ibrutinib [219]	Indoximod	-	Btk	NCT05106296	1	Ongoing

## Abbreviations

A172	human glioblastoma cells
A549	lung carcinoma cells
AC220	quizartinib
ALK	anaplastic lymphoma kinase
Akt	protein kinase B
BCL-2	B-cell lymphoma 2
BS-125	human glioblastoma cell line
BS-287	human glioblastoma cell line
Crk-II	CT10 regulator of kinase
CXCL	C-X-C motif chemokinel
DBTRG	human glioblastoma cell line
EBC1	lung squamous carcinoma cells
EGF	epidermal growth factor
EML4	echinoderm microtubule-associated protein-like 4
EOC2	mouse microglial cell lines
ERK	extracellular signal-regulated kinase
FGFR	fibroblast growth factor receptor
FLT3	Fms Related Receptor Tyrosine Kinase 3
Fyn	Src family tyrosine kinase
GBM6	human glioblastoma multiforme primary tissue cell line
GBM12	TMZ-resistant GBM cell line
GBM8401	human glioblastoma multiforme cell line
GBMSC83	glioblastoma stem cell line
GL261	glioma stem cell line
GSC11	glioma stem cell line
GSC20	glioma stem cell line
GSC23	glioma stem cell line
GSC407	glioma stem cell line
GSC923	glioma stem cell line
GSCD317	glioma stem cell line
HCC	hepatocellular carcinoma
HER	human epidermal growth factor receptor
HGK	hepatocyte progenitor kinase-like/germinal center kinase-like kinase
HUVECs	human umbilical vein endothelial cells
IGF	Insulin-like growth factor
JAK	Janus kinase
Kit	human tyrosine kinase receptor
LN-18	epithelial-like cell line
LN-229	epithelial-like cell line
MAPK	mitogen activated protein kinases
MCT	mast cell tumor
MDA-MB-231	epithelial, human breast cancer cell line
MKN45	gastric adenocarcinoma cells
MMP2	matrix metalloproteinase-2
mTOR	mammalian target of rapamycin
NF-κB	nuclear factor κB
PDA	pancreatic ductal adenocarcinoma cells
PDGF	platelet-derived growth factor
PI3K	phosphoinositide 3-kinase
PIK3R3	phosphoinositide-3-kinase regulatory subunit 3
Raf	rapidly accelerated fibrosarcoma
Ras	rat sarcoma
RET	rearranged during transfection tyrosine kinase receptor
RON	receptor tyrosine kinase
RT-PCR	Real time polymerase chain reaction
S6K	p70 ribosomal S6 kinase
siRNA	small interference RNA
SF-126-TR	high-grade human glioma cells temozolomide resistant
SF-539	human glioblastoma cell line
SF-767	human glioblastoma cell line
STAT3	signal transducer and activator of transcription 3
Tie-2	Angiopoietin-1 receptor tyrosine kinase
TGF-α	transforming growth factor-α
T98G	fibroblast-like cells
T98MG	human glioblastoma cancer cells
TGF-α	transforming growth factor alpha
U118MG-TR	U118 malignant glioma cells temozolomide resistant
U251	human glioblastoma cancer cells
U251-luc	transformed U251 human glioblastoma cancer cells
U373	uppsala human glioblastoma astrocytoma cell line
U87MG	uppsala 87 malignant glioma cell line
U87TxR	multidrug resistant uppsala 87 glioma cell line
uPA	urokinase-type plasminogen activator
uPAR	urokinase-type plasminogen activator receptor
VEGF	vascular endothelial growth factor
Yes	human non-receptor tyrosine kinase
